# Treatment of Chronic Bony Mallet Fingers by Dorsal Extension Block Pinning with Percutaneous Curettage

**DOI:** 10.1155/2018/7297951

**Published:** 2018-11-21

**Authors:** Fumiaki Takase, Kyoko Yamasaki, Takeshi Kokubu, Yutaka Mifune, Atsuyuki Inui, Ryosuke Kuroda

**Affiliations:** ^1^Department of Orthopaedic Surgery, Kobe University Graduate School of Medicine, 7-5-1 Kusunoki-cho, Chuo-ku, Kobe, Hyogo 650-0017, Japan; ^2^Department of Orthopaedic Surgery, Mitsubishi Kobe Hospital, 6-1-34 Wadamiya-Dori, Hyogo-ku, Kobe, Hyogo 652-0863, Japan

## Abstract

Dorsal extension block pinning is a commonly performed surgical technique for acute bony mallet fingers. However, the treatment of chronic bony mallet finger remains controversial. We investigated the use of dorsal extension block pinning with percutaneous curettage for chronic bony mallet fingers. Seven patients with chronic bony mallet fingers were treated by dorsal extension block pinning with percutaneous curettage. The average age was 17 (range, 12–23) years, and the average time from injury to surgery was 20 (range, 7–49) weeks. Bone union was achieved in all patients. None of the patients experienced pain after bone union. The average loss of distal interphalangeal joint extension was 6 (range, 5–20) degrees, and the average flexion was 59 (range, 40–80) degrees. The Crawford functional score was excellent in three patients, good in two, and fair in two. Dorsal extension block pinning with percutaneous curettage could be a useful treatment for chronic bony mallet fingers.

## 1. Introduction

Bony mallet finger is a common injuries of the hand which often occurs during a ball game. Some patients do not undergo prompt appropriate treatment or present to a hospital several weeks after the injury. If a bony mallet finger is not properly treated, it may become painful secondary to pseudoarthrosis and/or subluxation of the distal interphalangeal (DIP) joint. The extension block Kirschner wire (K-wire) technique, reported by Ishiguro et al. in 1997, is widely performed for treating acute bony mallet fingers [1]. This percutaneous method is less invasive compared with open reduction and internal fixation (ORIF) and is generally successful [2–4]. However, the treatment of chronic bony mallet fingers remains controversial. Many cases are treated by ORIF [5, 6] or conservative methods [7, 8]. However, ORIF can cause serious damage on small bony fragments and may have skin complications [9, 10]. This study evaluated the use of dorsal extension block pinning with percutaneous curettage for treating chronic bony mallet fingers at least 5 weeks after the injury.

## 2. Methods

Seven patients with chronic bony mallet fingers were treated by dorsal extension block pinning with percutaneous curettage. The average age was 17 (range, 12–23). In this study, as a definition of chronic bony mallet fingers, we decided that the time from injury to surgery had to be >5 (average, 20; range, 7–49) weeks. All surgeries were performed under digital block anesthesia. Under image intensifier guidance, the fracture site was percutaneously curettaged using an 18 G needle for soft tissue removal and decortication. We took time to curettage the fibrotic tissue by applying the needle tip to the center of the digit and the fracture site of distal phalanx directly. The fracture site was reduced using a 1.0 or 1.2 mm K-wire that was inserted in the middle phalanx along the dorsal edge of the bone fragment with the DIP and proximal interphalangeal (PIP) joints flexed. To achieve reduction, the DIP joint was extended by applying finger traction and volar pressure to the base of the distal phalanx. Another 1.0 or 1.2 mm K-wire was used to temporarily immobilize the DIP joint. An aluminum splint was added with the DIP joint extended and the PIP joint flexed for 2 weeks. The PIP joint was flexed because the central slip of the extensor was being relaxed in this position [11]. Ishiguro et al. reported K-wires were removed at 4 weeks and removal was postponed by 1 week if there was a delayed union [1]. Because our cases were chronic series, both K-wires were removed at 5 weeks and the range of motion exercise was initiated. Night splint was used for a further 4 weeks. Physical work was permitted after the radiograph bone healing.

## 3. Results

No infections occurred during the follow-up period, and all patients achieved bone union by 12 (range, 10–14) weeks after the surgery. No patients experienced pain after bone union. The average extension loss of the DIP joint was 6 (range, 5–20) degrees, and the average flexion was 59 (range, 40–80) degrees. Lateral view of the radiographs showed an average articular step-off of 0.8 (range, 0.1–1.2) mm immediately after the surgery and 0.7 (range, 0.1–1.0) mm at the final follow-up. The Crawford score was excellent in three patients, good in two, and fair in two (Table 1).

## 4. Case Six (Figure 1)

A 12-year-old boy injured his left middle finger while playing dodgeball. He visited a local clinic at 12 weeks after the injury because of persistent pain and was referred to our hospital for further management. Radiographs revealed a bony mallet finger without bony union. Conservative treatment with an aluminum splint was initially performed because the epiphyseal line was not closed. At 30 weeks after injury, the bone had not united, and the pain persisted. Thus, dorsal extension block pinning with percutaneous curettage was performed. Both K-wires were removed at 5 weeks after the surgery, and the range of motion exercise was initiated. Bony union was achieved at 12 weeks after the surgery (42 weeks after injury). At 20 weeks after the surgery (50 weeks after the injury), the DIP joint extension loss was 0 degrees and flexion was 60 degrees. The Crawford score was excellent.

## 5. Case Seven (Figure 2)

A 16-year-old boy injured his right middle finger while playing handball. The injury was surgically treated at a hospital. The fracture did not unite after the first surgery, but he did not experience pain. At 33 weeks after surgery, he began to experience pain while playing handball and was referred to our hospital for further management at 48 weeks after the surgery. Radiographs revealed a chronic bony mallet finger. Surgery was performed by dorsal extension block pinning with percutaneous curettage at 49 weeks after the injury. Both K-wires were removed at 5 weeks after the second surgery, and the range of motion exercise was initiated. Bony union was achieved at 10 weeks after the surgery (59 weeks after the injury). Radiographs revealed mild osteoarthritis of the DIP joint at 50 weeks after the surgery (99 weeks after the injury). The DIP joint extension loss was 20 degrees; flexion was 70 degrees. The Crawford score was fair.

## 6. Discussion

There are many reports on the treatment for chronic tendon mallet fingers or deformity [12, 13], but there are only a few reports on the treatment of chronic bony mallet fingers. Dorsal extension block pinning is widely performed to treat acute bony mallet fingers. It is also useful for treating bony mallet fingers within 5 weeks after injury; however, ORIF are recommended in chronic cases because satisfactory reduction is difficult to achieve if fibrous tissue is present at the fracture site. During open surgery, the surgeon can perform curettage under direct visualization. Lee et al. reported good results using ORIF with transtendinous wiring for patients having chronic bony mallet fingers [14]. However, the risks of breaking bone fragments, adhesion of extensor tendons, deformation of nails, or skin irritation are greater with open surgery than with closed surgery [15].

There are few reports regarding dorsal extension block pinning for treating chronic bony mallet fingers. Pegoli et al. reported six cases, with fair outcomes in three patients, good outcomes in two, and an excellent outcome in one [2]. Asano et al. reported 11 cases of chronic bony mallet fingers that were treated by dorsal extension block pinning and found that closed reduction by dorsal extension block pinning increased the risk for residual displacement and that a 0.5 mm step-off of the articular surface was gradually remodeled [16]. In our patients, dorsal extension block pinning was performed with percutaneous curettage; improvement of step-off was observed at the final follow-up. Patients with large extension loss of DIP joint before surgery tended to remain even at the final follow-up (Table 1).

Percutaneous curettage at the fracture site has benefits that include reducing the gap at the fracture site by removing scar tissues, with minimal damage of the skin and tendons, which helps prevent skin complications. However, not all cases can be treated in this manner. If palmar subluxation of the distal phalanx cannot be anatomically reduced, open reduction should be considered to avoid secondary osteoarthrosis [17]. In case seven, the interval from the initial injury may have affected clinical results, which supports prompt surgery even in chronic cases. Patient age may also affect the clinical outcome. In this study, all patients were young; most of them were teenagers. Active callus formation and the presence of a thick, tough periosteum promote rapid fracture healing in children [18] and good clinical outcomes. Dorsal extension block pinning can be the first choice of treatment for chronic bony mallet fractures in young patients. Because of the small number of patients evaluated, we could not compare the clinical outcomes of percutaneous curettage with dorsal extension block pinning with those of ORIF or dorsal extension block pinning only. Further study is required to compare these methods.

## Figures and Tables

**Figure 1:**
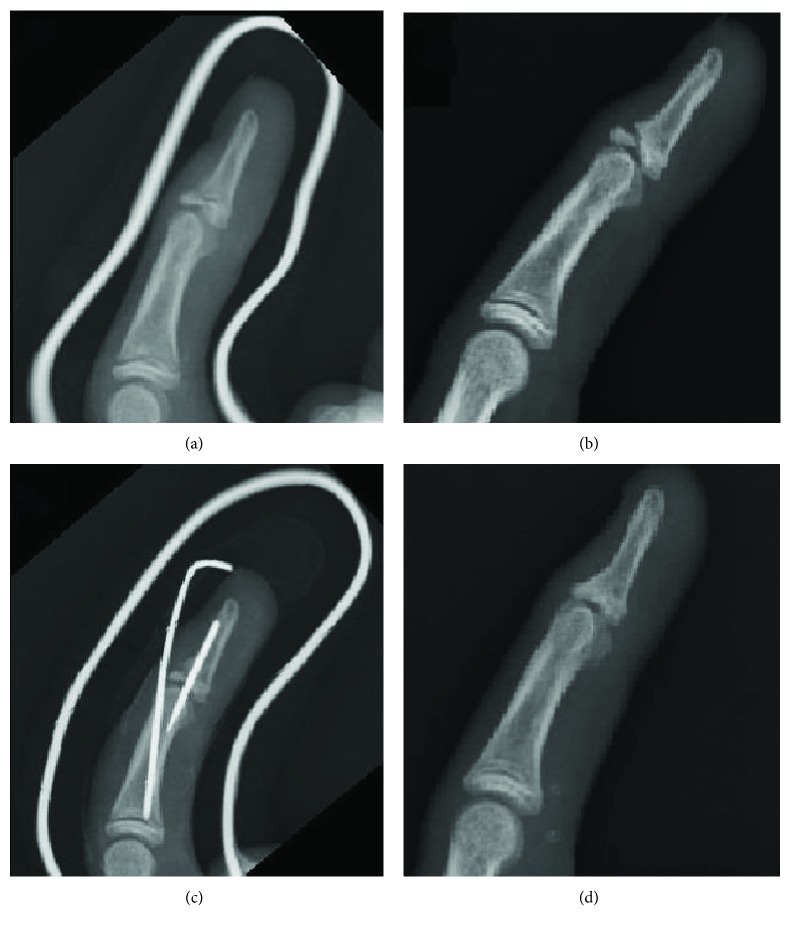
Case 6: lateral view radiographs of a 12-year-old boy. (a) Preoperative radiograph at 12 weeks after injury. (b) Bone union was not achieved after conservative treatment with an aluminum splint. (c) Postoperative radiograph at 30 weeks after injury. (d) The Crawford score was excellent at 50 weeks after the surgery.

**Figure 2:**
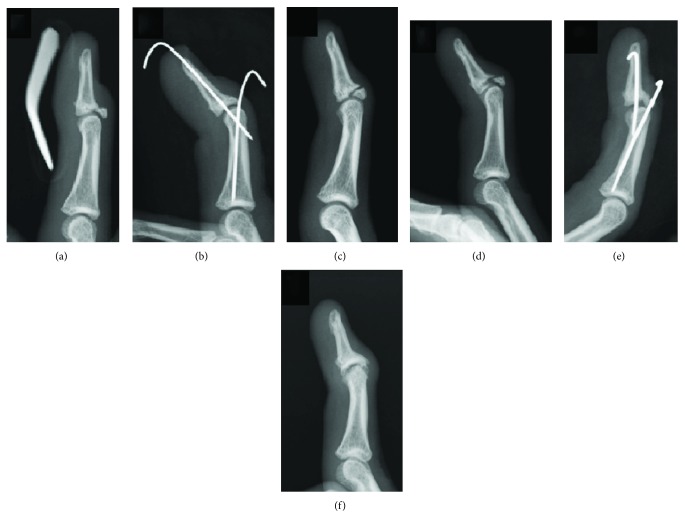
Case 7: lateral view radiographs of a 16-year-old boy. (a) Radiograph of the injury. (b) Radiograph after the previous surgery at another hospital. (c) Bone union was not achieved 10 weeks after the initial surgery. (d) Radiograph at 33 weeks showing the dislocation of a bone fragment. (e) Postoperative radiograph at our hospital at 49 weeks after the injury. (f) Bone union was achieved at 50 weeks after the second surgery. Although bone union was achieved, extension loss persisted. The radiograph shows mild osteoarthritis of the DIP joint.

**Table 1:** 

Case	Sex	Age (years)	Time from injury to operation (weeks)	Follow-up (weeks)	Extension loss (preoperative)	Extension loss (postremoval wire)	Extension/flexion (final follow-up)	Step-off post-Op (mm)	Step-off final follow-up (mm)	Crawford score
1	M	16	7	12	20	10	−15/40	0.1	0.1	Fair
2	F	22	7	12	5	5	0/50	0.8	0.6	Excellent
3	M	23	15	20	5	5	0/45	1	0.7	Good
4	F	16	16	73	20	15	−5/80	1.2	1.0	Good
5	M	16	17	12	5	5	0/70	0.5	0.5	Excellent
6	M	12	30	20	0	0	0/60	1.0	1.0	Excellent
7	M	16	49	50	30	30	−20/70	1	0.7	Fair
